# Silica Particles Functionalization with Thymol and Eugenol as a Novel Strategy to Control Histamine-Producing Bacteria

**DOI:** 10.3390/foods15122067

**Published:** 2026-06-08

**Authors:** Oumaima Moumane, Alejandro Rivas, Ana Fuentes, José Manuel Barat, Édgar Pérez-Esteve

**Affiliations:** Instituto Universitario de Ingeniería de Alimentos-Food UPV, Universitat Politècnica de València, Camino de Vera s/n, 46022 Valencia, Spain; oumou@etsiamn.upv.es (O.M.); alriso@upvnet.upv.es (A.R.); anfuelo@upv.es (A.F.); jmbrat@tal.upv.es (J.M.B.)

**Keywords:** histamine, essential oil components, immobilization, antimicrobial activity, food safety

## Abstract

Histamine accumulation in foods poses significant public health risks, yet current control strategies present important limitations. This study assessed the in vitro antimicrobial activity of silica microparticles covalently functionalized with thymol (SiO_2_-THY) and eugenol (SiO_2_-EUG) against four representative histamine-producing bacterial strains: *Raoultella planticola*, *Limosilactobacillus reuteri*, *Levilactobacillus hilgardii*, and *Pediococcus parvulus*. For this purpose, functionalized particles were characterized by FESEM, zeta potential measurements, and elemental analysis, confirming successful immobilization of both compounds at comparable functionalization degrees (137–139 mg EOC/g SiO_2_). Functionalized materials exhibited significant antimicrobial activity against all tested strains, with reductions exceeding 3 logarithmic cycles at the lowest concentrations tested. This effect is attributed to the functionalized particles altering bacterial membrane fluidity and disrupting membrane potential, thereby impairing cellular homeostasis, as revealed by mechanistic assays. Among the strains confirmed as histamine producers, SiO_2_-THY and SiO_2_-EUG substantially reduced histamine formation, with reductions reaching up to approximately 100% at bactericidal concentrations and remaining significant at sub-inhibitory levels. These findings suggest that immobilized compounds interfere not only with bacterial growth but also with histamine biosynthetic pathways. Therefore, the use of silica-immobilized essential oil constituents represents a promising strategy for mitigating histamine accumulation and enhancing food safety. Nevertheless, further validation in relevant food matrices is required.

## 1. Introduction

Biogenic amines (BAs) are nitrogenous compounds that may accumulate in foods as a consequence of microbial metabolic activity and are recognized as significant hazards for food safety and quality [1,2]. Among them, histamine is the most clinically relevant due to its direct implication in foodborne intoxications [3]. Although histamine plays essential physiological roles in the human body, excessive dietary intake can overwhelm detoxification mechanisms and pose a serious health risk [4].

High histamine levels are primarily associated with fish and fishery products, particularly those rich in free histidine, but also with fermented foods including meat products, cheese, vegetables, and alcoholic beverages [1,2]. In these matrices, histamine formation results from bacterial decarboxylation of histidine via histidine decarboxylase, a pyridoxal-5′-phosphate-dependent enzyme encoded within the histidine decarboxylase (HDC) gene cluster [5].

Within the European Union, histamine is subject to specific regulatory limits due to its recognized public health relevance. Maximum permitted levels are established under Commission Regulation (EC) No 2073/2005 using microbiological criteria for foodstuffs [6], which defines acceptable histamine concentrations in fishery products. Scientific risk assessments conducted by the European Food Safety Authority (EFSA) have further emphasized the importance of preventive measures to control histamine formation along the food chain [2,4]. In addition, surveillance data from the Rapid Alert System for Food and Feed (RASFF) indicate that histamine remains one of the most frequently notified chemical hazards in fish and fishery products within the European Union [7]. Such notifications often result in product recalls, batch rejections, and commercial losses, highlighting both the public health and economic impact of inadequate control. In fermented foods, although harmonized limits are not always defined, excessive histamine levels may compromise product safety, quality perception, and commercial value [8].

A wide range of bacteria have been identified as histamine producers in different food ecosystems. In fish and other protein-rich foods, members of the Enterobacteriaceae such as *Raoultella planticola* are frequently implicated in histamine accumulation [9,10,11]. In fermented dairy products and alcoholic beverages, several lactic acid bacteria including *Limosilactobacillus reuteri*, *Levilactobacillus hilgardii*, and *Pediococcus parvulus* have been shown to harbor the histidine decarboxylase gene cluster and actively produce histamine during fermentation [12,13]. Notably, histamine production is part of an amino acid decarboxylation system that contributes to intracellular pH homeostasis by consuming protons, thereby enhancing bacterial survival under acidic stress conditions typical of fermented foods [14]. Therefore, effective mitigation strategies must not only limit bacterial growth but also interfere with histamine-forming metabolic pathways.

Current control measures are mainly preventive and include strict temperature management along the cold chain, hygiene control, good manufacturing practices, and the use of starter cultures lacking decarboxylase activity [4,15]. While essential, these strategies present limitations. Temperature abuse may still occur during storage and distribution, starter culture selection does not eliminate contamination by indigenous microbiota, and fermentation processes inherently involve microbial metabolism, allowing histamine accumulation even under controlled technological conditions [16].

Natural antimicrobial compounds, particularly essential oil constituents (EOCs), have demonstrated broad-spectrum antimicrobial activity [17,18]. Several studies have reported their capacity to reduce biogenic amine accumulation mainly through inhibition of histamine-producing bacteria rather than through direct degradation of histamine. For instance, Ruiz-Rico et al. [19] demonstrated that carvacrol, thymol, eugenol and nisin significantly reduced histamine formation by histamine forming bacteria isolated from cheese, with complete inhibition observed at bactericidal concentrations and marked reductions at sub-bactericidal levels. These findings confirm the strong correlation between bacterial growth dynamics and histamine accumulation, highlighting microbial inhibition as a key preventive strategy. However, despite their demonstrated efficacy, the direct incorporation of essential oil components into food matrices remains limited by volatility, hydrophobicity, instability, and potential sensory impact [20,21].

Among the wide variety of EOCs, eugenol and thymol were selected for the present study. Eugenol (4-allyl-2-methoxyphenol) is a phenylpropanoid abundant in clove and other spices [18], whereas thymol (2-isopropyl-5-methylphenol) is a monoterpenoid phenol characteristic of *Thymus* and *Origanum* species [22]; both are widely used flavoring agents and are generally recognized as safe (GRAS) for food applications. Their antibacterial action is primarily attributed to the free phenolic hydroxyl group, which promotes partition into the lipid bilayer, disruption of membrane integrity, and dissipation of ion gradients. Carvacrol, a positional isomer of thymol [17], exhibits a comparable phenolic mechanism and potency; however, eugenol and thymol were prioritized here because they represent two distinct chemical classes (a monoterpenoid phenol and a phenylpropanoid), allowing the influence of molecular structure on immobilization and antimicrobial performance to be compared.

To overcome the limitations associated with the direct incorporation of EOCs into food matrices, covalent immobilization strategies have been increasingly explored [23,24,25]. Among the available carriers, silica-based materials represent particularly attractive supports due to their high surface area, chemical stability, mechanical resistance, and ease of surface functionalization. Importantly, these systems maintain antimicrobial efficacy without negatively affecting organoleptic properties, overcoming one of the major drawbacks of free EOC application [26,27]. However, the specific application of immobilized EOCs as a preventive strategy targeting histamine-producing bacteria and limiting histamine formation has not been systematically investigated.

In this context, the present study aimed to evaluate the antimicrobial potential of silica particles functionalized with thymol and eugenol against representative histamine-producing bacterial strains, namely *R. planticola*, *L. reuteri*, *L. hilgardii*, and *P. parvulus*. First, the effect of functionalized particles on bacterial growth was evaluated, together with their impact on cell integrity and physiological function, through the analysis of membrane fluidity, and membrane potential. Subsequently, the ability of the functionalized particles to reduce histamine formation was further assessed.

## 2. Materials and Methods

### 2.1. Materials

Thymol (THY, ≥98.5% *w*/*w*), eugenol (EUG, 99% *w*/*w*), (3-aminopropyl)triethoxysilane (APTES), Laurdan (6-dodecanoyl-2-dimethylaminonaphthalene), 3,3′-dipropylthiadicarbocyanine iodide (DiSC_3_(5)), pyridoxal-5′-phosphate (PLP), L-histidine, dansyl chloride (DNS-Cl), dimethyl sulfoxide (DMSO), disodium phosphate (Na_2_HPO_4_), potassium dihydrogen phosphate (KH_2_PO_4_), and silica particles (SiO_2_, 5–25 µm) were purchased from Sigma-Aldrich^®^ (Merck KGaA, Darmstadt, Germany). Acetone and ethanol were obtained from Labbox (Barcelona, Spain). Nutrient broth (NB), Man–Rogosa–Sharpe (MRS) agar and broth, peptone water, formaldehyde solution (37%, *v*/*v*), hydrochloric acid (HCl), sodium carbonate, diethyl ether, acetonitrile (HPLC grade), sodium chloride (NaCl), and potassium chloride (KCl) were supplied by Scharlab (Barcelona, Spain). Ammonium hydroxide solution (H_5_NO, 35%, *v*/*v*) was obtained from Thermo Scientific (Fisher Scientific, Madrid, Spain). Phosphate-buffered saline (PBS) was prepared in-house using analytical-grade reagents. The bacterial strains *R. planticola* CECT 843, *L. reuteri* CECT 925, *P. parvulus* CECT 4693, and *L. hilgardii* CECT 4681 were obtained from the Spanish Type Culture Collection (CECT, Valencia, Spain).

### 2.2. Synthesis of the Silica Microparticles Functionalized with Antimicrobial Compounds

**Figure 1:**
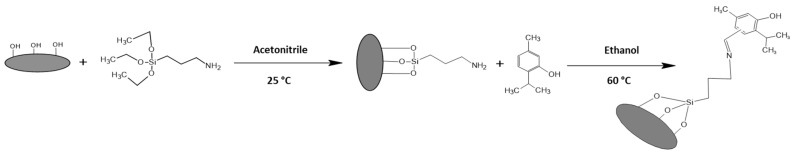
Representation of the synthesis procedure of the antimicrobial particles (SiO_2_-THY).

Silica particles (SiO_2_) were functionalized with EOCs following the procedure described by Rivas et al. [24] o synthesize the aldehyde group, with minor modifications. The overall synthesis route of the antimicrobial devices is schematically depicted in Figure 1. Briefly, 2 g of SiO_2_ particles (5–25 µm) were first reacted with 8.54 mmol of APTES and 25 mL of acetonitrile. The mixture was stirred at room temperature for 24 h. Subsequently, the mixture was centrifuged (11,000× *g*, 5 min) to separate the solid fraction, which was dried under vacuum for 24 h. Several washing steps were carried out, first with acetonitrile, followed by washing with distilled water at pH 4 to remove free APTES and neutralize the reaction. For the covalent anchoring of EOCs to SiO_2_-APTES particles, 2.14 mmol of formaldehyde and 8.54 mmol of THY or EUG were added to 50 mL of ethanol in a round-bottom flask and stirred at 60 °C for 24 h. Afterwards, the mixture was subject to centrifugation (11,000× *g*, 5 min) and the solid phase obtained was submitted to different washes. The first washes were performed with ethanol, followed by washes with water at pH 4. Finally, the functionalized particles were dried under vacuum for 24 h to obtain the solid SiO_2_-Thymol (SiO_2_-THY) or SiO_2_-Eugenol (SiO_2_-EUG).

### 2.3. Silica Particles Characterization

Bare and EOC-modified SiO_2_ particles were systematically characterized using complementary analytical approaches to evaluate their morphology, surface charge behavior, and grafting efficiency. Morphological features were examined by Field-Emission Scanning Electron Microscopy (FESEM). Micrographs were collected using a Zeiss Ultra 55 microscope (Carl Zeiss NTS GmbH, Oberkochen, Germany) operating in secondary electron mode, allowing detailed visualization of particle surface topology and structural integrity after functionalization. Surface charge properties were assessed through zeta potential (ζ) measurements using a Zetasizer Nano ZS (Malvern Instruments, Worcestershire, UK). Electrophoretic mobility values were converted into ζ potential using the Smoluchowski approximation. All measurements were conducted at 25 °C and performed in triplicate to ensure reproducibility. The extent of organic functionalization was quantified by CHN elemental analysis employing a Vario EL III elemental analyzer (Elementar Analysensysteme GmbH, Langenselbold, Germany). Elemental analysis enabled the determination of carbon content, which was used to calculate the degree of functionalization (α), expressed as mg of organic material (OM) per gram of silica. These values were subsequently converted into mmol/g using the molecular weight of the immobilized compounds [28]. To account for differences in textural properties, the grafted amount was normalized by the specific surface area (SSA) obtained from BET analysis, allowing the calculation of EOC surface density (µmol/m^2^). Based on this parameter, the surface modification efficiency was further evaluated using global surface coverage and APTES-relative coverage. These descriptors provide complementary information on the extent of surface occupation and the efficiency of the coupling reaction. All calculations were performed according to the methodology described by Moumane et al. [25].

### 2.4. Microbiological Assays

The bacterial strains were reconstituted according to the CECT guidelines, and the bacterial stocks were stored at 4 °C in the appropriated culture medium until use. For microbiology experimental assays, a single colony of each strain was selected and inoculated into the corresponding culture medium. *R. planticola* was grown aerobically on NA at 30 °C for 24 h, whereas *L. reuteri* was cultured aerobically in MRS medium at 37 °C for 24 h. Finally, *P. parvulus* and *L. hilgardii* were grown under microaerophilic conditions in MRS medium at 37 °C for 48 h.

#### 2.4.1. Determination of the Minimum Inhibitory Concentration (MIC) and Minimum Bacterial Concentration (MBC) of the Free EOCs

The susceptibility of the different bacteria to THY and EUG was evaluated using the broth microdilution method according to the CLSI M100 performance standards [29]. This procedure allowed the determination of the minimum inhibitory concentration (MIC), defined as the lowest antimicrobial concentration at which no visible microbial growth is observed after incubation and the minimum bacterial concentration (MBC), defined as the lowest concentration capable of killing 99.99% of the microbial population.

Stock solutions of the bioactive compounds were prepared by dissolving each compound in dimethyl sulfoxide (DMSO). To ensure sterility, the stock solutions were filtered through sterile 0.22 µm syringe filters prior to use.

Bacterial growth was monitored using sterile 96-well microplates. Wells were filled with 190 μL of the appropriate broth medium containing the antimicrobial stock solution to achieve final concentrations ranging from 0.1 to 4 mg/mL. Subsequently, 10 μL of bacterial inoculum adjusted to approximately 10^8^ CFU/mL was added to each well. The microplates were incubated under optimal growth conditions of temperature and incubation time for each strain. Two negative controls were included: one containing broth and DMSO without inoculum, and another containing the highest tested concentration of the antimicrobial compounds in broth to account for possible initial turbidity caused by the compounds in DMSO. Two positive controls were also included: well with broth and inoculum, with and without DMSO. Finally, absorbance at 595 nm was initially measured (0 h) and after the appropriate incubation period for each strain using a microplate reader. To determine the MBC, aliquots were taken from the wells that showed no visible turbidity and plated onto the appropriate culture medium for each strain. The plates were incubated at 37 °C for 24–48 h.

#### 2.4.2. Antibacterial Activity of Immobilized Antimicrobials

To evaluate the antimicrobial activity of the functionalized particles, sterile 1.5 mL microcentrifuge tubes containing 1 mL of culture broth were prepared. Particle concentrations equivalent to MIC×0.25, MIC×0.5, MIC and MIC×2 were tested, with the actual range of concentrations adjusted for each bacterial strain according to its MIC and the degree of particle functionalization. Subsequently, the microcentrifuge tubes were inoculated with 100 μL of inoculum adjusted to 10^6^ CFU/mL. The tubes were incubated under orbital shaking at the optimal growth temperature of each strain for 24 h or 48 h. After incubation, the tubes were centrifuged for 2 min to sediment the functionalized particles, while keeping the bacterial cells in suspension. Then, 100 μL of the supernatant was transferred to Petri dishes containing the appropriate growth medium for each strain and incubated under optimal conditions. The assays included two positive controls (one tube containing inoculum and culture medium bare particles, and another tube containing inoculum and culture medium with non-functionalized particles) and a negative control (tube containing culture medium without inoculum or particles).

### 2.5. Mechanism of Action of Free and Immobilized EOCs

To elucidate the mechanism of action of free and immobilized bioactive compounds, changes in membrane fluidity and membrane potential were evaluated using complementary fluorescence-based assays.

#### 2.5.1. Membrane Fluidity (Laurdan Generalized Polarization)

Membrane fluidity was evaluated using Laurdan (6-dodecanoyl-2-dimethylaminonaphthalene), a polarity-sensitive fluorescent probe that reports changes in lipid packing and bilayer organization through shifts in emission spectra [30].

Bacterial inocula were prepared at OD_600_ = 0.4 and exposed to treatments for the indicated times (15 min, and 24 h) at the optimal temperature for each strain. After treatment, cells were washed and resuspended in PBS. Laurdan (final concentration 10 µM) was added, and samples were incubated at room temperature in the dark for 30 min. Fluorescence emission was recorded at 440 nm and 490 nm (excitation 350 nm). All values were standarized using bare silica particles as a negative control to avoid possible interference with the Laurdan probe. Generalized Polarization (*GP*) values were calculated as previously described by Parasassi et al. [31]:

$$GP=\frac{I_{440\;nm}-I_{490\;nm}}{I_{440\;nm}+I_{490\;nm}}$$

Changes in *GP* values were interpreted as alterations in membrane order and lipid packing. Similar approaches have been widely applied to evaluate antimicrobial-induced membrane perturbations in bacteria [32].

#### 2.5.2. Membrane Potential (DiSC_3_(5) Assay)

Membrane potential (ΔΨ) was assessed using the voltage-sensitive probe DiSC_3_(5), a lipophilic cationic dye that accumulates in polarized bacterial membranes and undergoes fluorescence changes upon depolarization [33]. Bacterial suspensions (OD_600_ = 0.4) were incubated with DiSC_3_(5) (5 µM) at room temperature in the dark for 15 min until a stable baseline fluorescence was reached. Treatments were subsequently applied, and fluorescence was monitored in real time (excitation 622 nm; emission 670 nm), as previously described for antimicrobial-induced membrane depolarization studies [34].

Appropriate controls were included in all assays, including untreated cells (negative control) and cells exposed to membrane-disrupting agents (positive control). Immobilized particles without bacterial inoculum were also analyzed to exclude autofluorescence interference.

### 2.6. Evaluation of Histamine Formation by HPLC

Given that histamine production is a strain-dependent characteristic, only decarboxylase-positive strains previously identified as histamine producers were selected to evaluate the effect of the antimicrobial treatment. This approach ensured that any reduction in histamine accumulation could be directly associated with the inhibition of histamine-producing bacteria.

To determine whether functionalized particles were able to reduce histamine accumulation, histamine production was quantified by HPLC following dansyl chloride derivatization [35,36]. For this purpose, sterile 1.5 mL microcentrifuge tubes containing 1 mL of culture, culture broth and PLP, grape must, and grape must and PLP, all of them supplemented with 1% histidine to favor histamine production, were put in contact with immobilized particles at tested concentrations. Every tested condition was conducted both at the original pH of each medium and after adjustment to pH 4.2 to evaluate the influence of pH and cofactor availability on histamine production. Each tube was then inoculated with 100 µL of inoculum adjusted to a concentration of 10^6^ CFU/mL. The tubes were then incubated for 48 h at the optimal temperature for each strain studied.

After incubation, the samples were centrifuged, and the supernatant was acidified by adding 22 µL of 0.1 M HCl, followed by chemical derivatization. The dansylated derivatives were formed by adding to 1 mL of culture broth, 300 µL of a saturated Na_2_CO_3_ solution, and 1 mL of dansyl chloride solution (DNS-Cl, 10 mg/mL in acetone). The samples were vortexed for 1 min and incubated in the dark at 60 °C for 30 min. To stop the reaction, 100 µL of H_5_NO (25% *v*/*v*) was added and left to stand at room temperature in darkness for 15 min. Afterwards, 2.5 mL of diethyl ether was added to extract the biogenic amines, and the mixture was homogenized, and frozen at −40 °C. The organic phase was recovered in borosilicate tubes, and the extraction was repeated twice, yielding a total of 5 mL of organic phase. The organic phase was concentrated under a stream of nitrogen (N_2_) and the resulting dry residue was dissolved in 0.5 mL of acetonitrile and filtered through a 0.45 µm filter. The filtered extract was transferred to amber vials for HPLC analysis.

The analysis was performed using a Hitachi LaChrom Elite HPLC system (Hitachi Ltd., Tokyo, Japan) equipped with an autosampler (model L-2200) and a UV detector (model L-2400). Chromatographic separation was carried out on a ZORBAX Eclipse XDB-C18 (150 mm × 4.6 mm, internal diameter 5 μm, Agilent Technologies, Santa Clara, CA, USA). The mobile phase consisted of an isocratic mixture of acetonitrile (phase A) and Milli-Q water (phase B) for 15 min, with a flow rate of 0.8 mL/min at 25 °C. The injection volume was 10 µL, and UV detection was performed at 254 nm.

The validation of the chromatographic method was previously performed according to the guidelines laid down in Directive 96/23/CE. The technical validation parameters were calculated in terms of linearity, limit of detection (LOD), limit of quantification (LOQ), precision, accuracy, and recovery. The results of the method validation are shown in Table S1 (Supplementary Materials). Briefly, the method exhibited satisfactory linearity over the working range, together with LOD, LOQ, precision, accuracy, and recovery values within the limits required for the reliable quantification of histamine in the studied media (Table S1).

### 2.7. Statistical Analysis

Prior to analysis, the assumptions of the parametric tests were verified: the normality of the residuals was assessed using the Shapiro–Wilk test, and the homogeneity of variances was checked using Levene’s test. When these assumptions were met, the influence of the different EOCs and the concentrations of the immobilized EOCs on bacterial viability was analyzed by a multifactor analysis of variance (ANOVA), and the LSD (least significant difference) procedure was used to test differences between means at the 5% significance level.

## 3. Results and Discussion

### 3.1. Determination of the MIC and MBC of Thymol and Eugenol

Table 1 shows the MIC and MBC values obtained for each bioactive compound against the tested strains (*R. planticola*, *L. reuteri*, *P. parvulus*, and *L. hilgardii*). Overall, THY exhibited greater antimicrobial activity than EUG across all strains.

Among the tested microorganisms, *R. planticola* was the most susceptible strain, with MIC values of 0.3 mg/mL for THY and 1 mg/mL for EUG, and MBC values of 0.4 and 1.1 mg/mL, respectively. In contrast, *L. reuteri* displayed the highest tolerance, requiring 1 mg/mL of THY and 3.5 mg/mL of EUG to inhibit growth, with similar MBC values. *P. parvulus* and *L. hilgardii* showed intermediate susceptibility, with MIC values ranging from 1 to 1.5 mg/mL and MBC values from 2 to 4 mg/mL for thymol, whereas for eugenol, MIC values were 2 mg/mL and MBC values ranged from 2.5 to 4 mg/mL.

These results are consistent with previously reported data on the antimicrobial activity of phenolic essential oil compounds. THY and carvacrol have shown inhibitory activity against various bacteria, with MIC values typically between 0.25 and 2 mg/mL depending on the strain and experimental conditions [18,19,37]. Similarly, EUG has demonstrated inhibitory activity against different bacterial strains, although MIC values vary depending on the strain and experimental conditions [38,39]. The values obtained for *L. reuteri*, *P. parvulus*, and *L. hilgardii* therefore fall within these previously described ranges. Minor differences between studies may reflect strain-specific susceptibility, inoculum density, growth phase, and methodological variations [17].

Regarding the higher activity of THY over EUG, this trend aligns with studies comparing structurally related phenolic compounds. For instance, a recent study on *Klebsiella pneumoniae* reported MIC values of 0.26 mg/mL for EUG and 0.12 mg/mL for carvacrol, confirming the greater antimicrobial potency of carvacrol [40]. Since *R. planticola* was previously classified within the genus *Klebsiella*, and THY is an isomer of carvacrol, these findings support both the susceptibility pattern and the compound ranking observed in the present study.

Finally, the low MIC and MBC values obtained for *R. planticola* (0.3 and 0.4 mg/mL for THY; 1 and 1.1 mg/mL for EUG) are consistent with the ranges reported in the literature for phenolic monoterpenes against Gram-negative and Gram-positive bacteria [18,19,37] suggesting comparable sensitivity to these compounds. Overall, these results are in line with the proposed mechanism of action of thymol and eugenol, which involves disruption of the cell membrane and alteration of its permeability.

### 3.2. Characterization of Functionalized Materials

**Figure 2:**
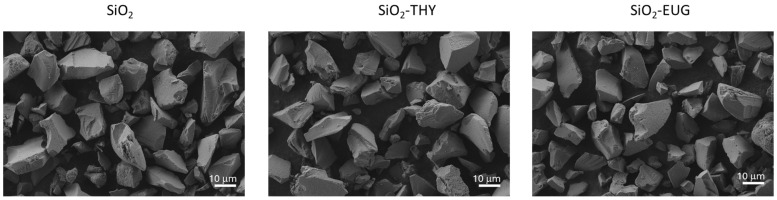
FESEM images of the bare and functionalized SiO_2_ particle at 1000×.

Before conducting the antimicrobial assays with immobilized EOCs, the bare SiO_2_ particles and those functionalized with thymol (SiO_2_-THY) or eugenol (SiO_2_-EUG) were subjected to physicochemical characterization using standard analytical techniques. FESEM images show no appreciable alterations in particle size, geometry or surface topography after immobilization (Figure 2). These observations indicate that the functionalization process did not compromise the structural features of the silica supports, which is consistent with previous reports on covalent grafting of organic molecules onto silica-based carriers [24,25].

Zeta-potential (ζ) measurements were performed for both silica particles to assess surface charge modification after immobilization (Table 2). Both bare silica particles and those functionalized with THY and EUG showed zeta potential values within the range of −30 mV to +30 mV, suggesting moderate particle stability. Bare silica exhibited a negative ζ (−27.7 mV), consistent with the presence of deprotonated silanol groups. After functionalization, all samples showed a marked shift toward positive values, indicating the successful grafting of amino and phenolic groups. ζ increased to +0.1 ± 1.6 mV for SiO_2_-Thy and +3.8 ± 0.8 mV for SiO_2_-EUG. These results are consistent with those reported in previous works on silica functionalization [25]. This shift further supports the effective immobilization of the bioactive compounds.

Table 2 also summarizes the functionalization parameters of silica particles modified with thymol and eugenol, including the degree of functionalization (α), EOC content (mg EOC/g SiO_2_), APTES-relative coverage and global EOC surface coverage. The EOC content, expressed as mg of immobilized compound per gram of silica, was derived from elemental (C, H, N) analysis to estimate the functionalization yield and assess grafting efficiency. Elemental quantification is commonly used to determine organic incorporation onto inorganic supports and has been adapted for evaluating the immobilization of essential-oil-derived compounds. Both functionalized particles exhibited comparable α values, reaching 136.9 mg OM/g SiO_2_ for SiO_2_-THY and 139.1 mg OM/g SiO_2_ for SiO_2_-EUG, indicating similar overall incorporation of organic matter onto the particle surface. The majority of this organic matter corresponded to APTES, while the amount of EOC immobilized on the silica particles was approximately the same for both compounds (ca. 26–28 mg EOC/g SiO_2_). This yielded an almost identical surface coverage for both (ca. 4.5%), suggesting that thymol and eugenol were successfully immobilized to a comparable extent. The slightly higher incorporation observed for SiO_2_-EUG, although moderate, may reflect differences in molecular structure and reactivity, consistent with findings reported by Gómez-Llorente et al. [27].

### 3.3. Antibacterial Activity of Functionalized Supports

**Figure 3:**
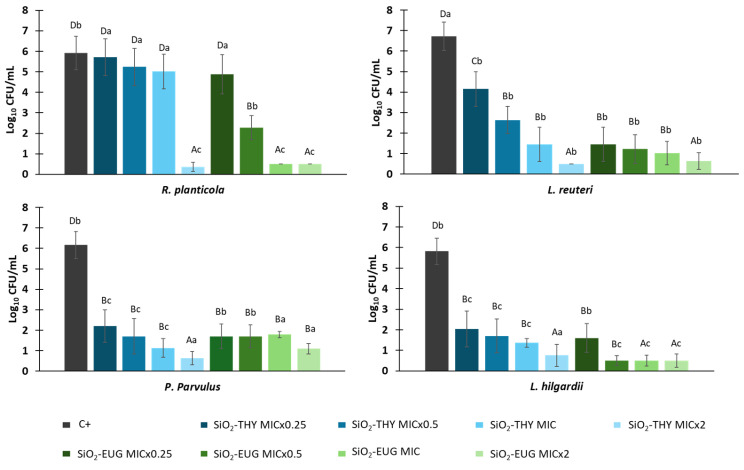
Effect of bioactive compounds immobilized on silica particles on histamine-producing strains. Values are expressed as mean ± SD (n = 3). Different uppercase letters indicate significant differences between treatments within the same bacterial strain, while different lowercase letters indicate significant differences between bacterial strains within the same treatment (*p* < 0.05).

As no previous data are available regarding the antimicrobial performance of SiO_2_-EOC supports against histamine-producing bacteria under in vitro culture conditions, preliminary assays were conducted in growth media to evaluate their intrinsic antimicrobial activity. Figure 3 shows the effect of SiO_2_-Immobilized THY and EUG on the growth of the four tested histamine-producing bacteria. Overall, the presence of functionalized particles resulted in a significant reduction in bacterial growth in all evaluated strains, with immobilized EUG generally exhibiting the strongest antimicrobial effect. It is worth noting that immobilization of bioactive compounds derived from essential oils may enhance their antimicrobial activity, possibly due to the higher local concentration of the antimicrobial agent achieved when anchored onto silica surfaces, which promotes direct interaction with bacterial cells [41]. This behavior may explain the high efficacy observed in several of the tested treatments.

In the case of *R. planticola*, both functionalized compounds showed notable efficacy, significantly reducing bacterial growth. This reduction became more pronounced as the concentration of the active compound increased, particularly under the MIC×2 condition for thymol. However, for immobilized eugenol, bacterial growth was almost completely suppressed even at lower concentrations.

*L. reuteri* exhibited high sensitivity to the tested treatments, with a clear decrease in bacterial growth observed from concentrations equivalent to the MIC, with eugenol again showing the strongest inhibitory effect.

Regarding *P. parvulus* and *L. hilgardii*, both microorganisms also showed significant growth reduction. Notably, even at concentrations equivalent to MIC×0.25 of the functionalized compounds, a reduction greater than three logarithmic cycles was observed, indicating high sensitivity of these strains to the evaluated treatments.

Comparison with the antimicrobial results obtained for the free compounds revealed a different antimicrobial pattern after immobilization. Free THY exhibited higher antimicrobial activity than free EUG against all tested strains, as reflected by its lower MIC and MBC values. In contrast, SiO_2_-EUG generally caused a similar or stronger reduction than SiO_2_-THY, particularly against *R. planticola*. This behavior is consistent with previous studies showing that the antimicrobial performance of immobilized EOCs does not necessarily mirror that of the corresponding free compounds [24,25]. Instead, the efficacy of immobilized systems appears to depend not only on the intrinsic antimicrobial potency of the molecule, but also on surface-related factors such as grafting efficiency, molecular accessibility, spatial presentation, local concentration at the particle–cell interface, and direct interaction with bacterial envelopes. Therefore, the enhanced performance of SiO_2_-EUG under the tested conditions may be associated with a more favorable surface presentation or accessibility of eugenol after immobilization.

### 3.4. Impact of Free and Immobilized Bioactive Compounds on Bacterial Membrane Function

**Figure 4:**
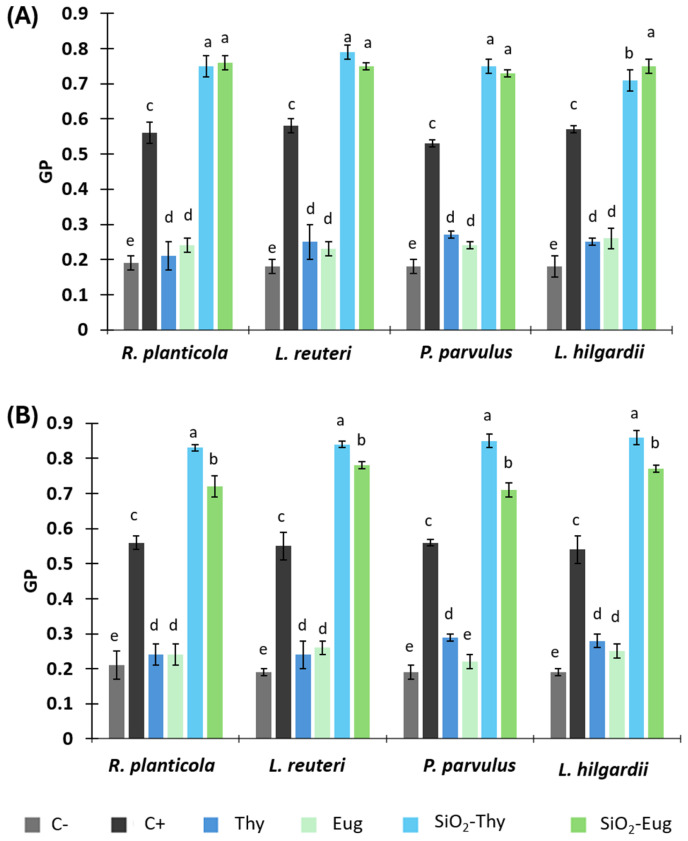
*GP* values of *R. planticola*, *L. reuteri*, *P. parvulus* and *L. hilgardii* cells (C+) treated with free (THY and EUG) or immobilized EOCs (SiO_2_-THY and SiO_2_-EUG) (**A**) 15 min and (**B**) 24 h. Values are expressed as mean ± standard deviation (n = 3). Different lowercase letters indicate significant differences among treatments for the same bacterial strain (*p* < 0.05).

In Figure 4, the effect of free and EOCs immobilized on SiO_2_ particles on bacterial membrane properties is presented as Generalized Polarization (*GP*) values. In all tested strains (*R. planticola*, *L. reuteri*, *P. parvulus*, and *L. hilgardii*), exposure to SiO_2_-THY and SiO_2_-EUG markedly increased *GP* values compared with the untreated control and the corresponding free EOCs. This increase suggests a reduction in membrane fluidity and a higher degree of lipid packing, indicating that immobilized phenolic compounds strongly affect bacterial membrane organization. EOs and their phenolic constituents, such as THY and EUG, have been widely reported to disrupt bacterial membranes by altering permeability and lipid organization, leading to leakage of intracellular components and loss of viability [42].

Therefore, the immobilization of THY and EUG on silica particles may promote a more localized and progressive interaction with the bacterial surface compared with free compounds. This could trigger membrane reorganization rather than immediate disruption, resulting in increased lipid ordering. Although this strategy may initially help bacteria cope with stress, the associated metabolic cost and altered membrane functionality could ultimately compromise cellular activity and viability. Such behavior has been reported in other antimicrobial systems where membrane rigidification precedes growth inhibition or cell death [43].

**Figure 5:**
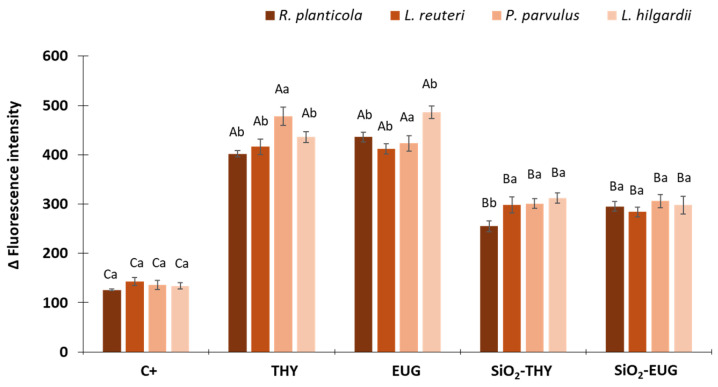
Membrane potential (ΔΨ) of *R. planticola*, *L. reuteri*, *P. parvulus* and *L. hilgardii* cells (C+) treated with free (THY and EUG) or immobilized (SiO_2_-THY and SiO_2_-EUG) EOCs. Values are expressed as mean ± standard deviation (n = 3). Different uppercase letters indicate significant differences among treatments for the same bacterial strain, while different lowercase letters indicate significant differences among bacterial strains within the same treatment (*p* < 0.05).

Figure 5 shows the results of the effect of bioactive compounds, both free and immobilized, on the cell membrane potential (Δψ). As observed, the contact of the bacterial cells of each studied strain with the tested antimicrobials produced an increase in fluorescence intensity, which indicates a loss of membrane potential. This effect was consistently observed in all tested strains, confirming that both compounds interfere with membrane electrochemical gradients. Due to their hydrophobic nature, THY and EUG can partition into the lipid bilayer, disrupting membrane integrity, increasing permeability, and altering ion gradient [44]. In the same way, silica-immobilized compounds (SiO_2_-THY and SiO_2_-EUG) also caused a clear increase in fluorescence compared to the control, confirming that the immobilized bioactive compounds retained their ability to disrupt membrane potential. However, the magnitude of depolarization was lower than that observed for the free compounds, likely due to restricted molecular mobility and the requirement for direct contact between the functionalized particles and the bacterial cell surface. This suggests that the antimicrobial effect of immobilized compounds may be more dependent on direct contact interactions, potentially leading to a more localized but controlled mode of action compared to free compounds.

### 3.5. Effect of Immobilized Bioactive Compounds on Histamine Formation

*L. reuteri* and *R. planticola* were selected to test the effect of SiO_2_-immobilized particles on histamine formation, as they represent two physiologically distinct groups: *L. reuteri* is a Gram-positive LAB associated with histamine accumulation in dairy products, while *R. planticola* is a Gram-negative enterobacterium responsible for histamine in fish products. *L. hilgardii* and *P. parvulus* were excluded from this phase of the study because histamine production by these wine-associated LAB is highly dependent on enological factors such as fermentation temperature, pH, ethanol content, and organic acid concentrations during malolactic fermentation [5], making it difficult to isolate the effect of silica particles on microbial growth and histamine accumulation. Furthermore, no detectable histamine production was observed for either species under the tested conditions, which may be explained by the absence or non-expression of the HDC gene cluster. In particular, the histidine decarboxylase pathway in *L. hilgardii* is encoded on an unstable plasmid that can be lost under certain conditions [13], and histamine production is known to vary considerably even among strains of the same species due to differences in the presence, integrity, or regulation of *hdc* [12,13]. Future work should then examine the effect of essential oils, their bioactive compounds, and their immobilization on silica particles on *hdc* expression and HDC activity.

The effect of functionalized particles on histamine formation *R. planticola* and *L. reuteri* is summarized in Table 3. The immobilized compounds significantly reduced histamine formation in both bacteria. In general, higher reductions were observed at increasing concentrations, suggesting a clear dose-dependent effect. In the case of SiO_2_-THY, *R. planticola* showed greater susceptibility to treatments with high levels of inhibition, even at concentrations below the MIC. In contrast, *L. reuteri* required a higher concentration to inhibit histamine formation. For SiO_2_-EUG particles, an even stronger inhibitory effect was observed at the lowest studied concentration (MIC×0.25), leading to a reduction of up to approximately 82% in the least susceptible strain (*R. planticola*).

The reduction in histamine formation observed can be linked to the antimicrobial activity of the compounds studied. For example, in the case of *R. planticola*, treatment with SiO_2_-THY at MIC×2 completely inhibited bacterial growth, which explains the absence of histamine production under these conditions. However, at lower concentrations (MIC, MIC×0.5, and MIC×0.25), viable bacterial cells were still detected, and histamine formation was reduced by up to 69.5%. A similar trend was observed for SiO_2_-EUG, where significant reductions in histamine levels were detected. A comparable behavior was observed in *L. reuteri*. Although growth inhibition was more pronounced at higher concentrations, THY-functionalized particles reduced histamine formation by 59.8–99.5%, while EUG-functionalized particles achieved reductions between 92.3% and 100%, even when viable cells were still viable. Therefore, the results indicate that immobilized compounds may also interfere with the metabolic pathways involved in histamine formation. This effect could also be related to alterations in membrane permeability (Section 3.4).

These findings suggest that immobilized EOCs may reduce histamine formation through a dual effect: limiting bacterial growth and impairing the metabolic processes involved in histidine decarboxylation. This interpretation is consistent with previous work on histamine-forming LAB, where free antimicrobial compounds reduced histamine production even at sub-inhibitory concentrations. In *L. parabuchneri* and *L. reuteri*, carvacrol at MBC×0.5 reduced histamine content by 88% and 96%, respectively, while eugenol at MBC×0.5 almost completely suppressed histamine generation in *L. reuteri* [20]. Moreover, Ruiz-Rico et al. [20] reported that carvacrol affected not only cell viability but also bacterial metabolism, as decarboxylase activity was modified by the presence of the antimicrobial compound. Therefore, the reduction in histamine observed here may involve interference with the histidine decarboxylase pathway, although this mechanism should be confirmed by specific assays evaluating *hdc* expression and HDC activity. In addition, the membrane alterations observed after exposure to the functionalized particles (Section 3.4) may contribute to this effect by disturbing membrane-associated transport processes, proton balance, or cellular homeostasis required for efficient histidine decarboxylation.

## 4. Conclusions

The results of this study demonstrate that silica microparticles covalently functionalized with thymol (SiO_2_-THY) and eugenol (SiO_2_-EUG) exert significant antimicrobial activity against all four tested histamine-producing strains (*R. planticola*, *L. reuteri*, *P. parvulus*, and *L. hilgardii*), with bacterial reductions exceeding three logarithmic cycles at concentrations as low as MIC×0.25. Mechanistic studies confirmed that both materials altered bacterial membrane fluidity and disrupted membrane potential, consistent with a progressive, surface-mediated mechanism of action. Notably, immobilized EUG generally outperformed immobilized THY in antimicrobial potency, a reversal relative to the free compounds, likely reflecting differences in surface orientation and membrane contact dynamics upon covalent grafting. Among the strains confirmed as histamine producers, both functionalized materials substantially reduced histamine formation in a dose-dependent manner, with reductions reaching up to approximately 100% at bactericidal concentrations and remaining significant at sub-inhibitory levels. These findings suggest two complementary mechanisms: a reduction in the viable histamine-producing population, and a potential direct interference with histamine biosynthetic pathways, possibly through impairment of cofactor availability or HDC enzyme activity, although the latter requires confirmation through dedicated gene expression and enzyme activity studies. Specifically, histamine reduction ranged from approximately 60% at the lowest sub-inhibitory concentration (MIC×0.25) to nearly complete inhibition (~100%) at bactericidal concentrations, with SiO_2_-EUG being particularly effective against both *R. planticola* and *L. reuteri*, reducing histamine formation by more than 92% even at MIC×0.25.

Overall, these results highlight the potential of silica-immobilized bioactive compounds as a promising strategy to control histamine-producing bacteria and mitigate histamine accumulation in food systems. This method effectively overcomes key limitations of free EOC application, including volatility, instability, and sensory impact. Considering the significant public health risks associated with histamine intoxication, the application of this approach could contribute to improving food safety and reducing the incidence of histamine-related foodborne illnesses.

Nevertheless, future work should validate the performance of these systems in real food matrices such as fish, cheese, and wine, and address regulatory and safety aspects related to particle migration and EOC residue levels prior to any practical application in the food industry. Moreover, it should be acknowledged that the present results were obtained under in vitro conditions and after relatively short incubation periods (24–48 h). Since histamine accumulation in foods is a time-dependent process that may proceed over several days or weeks during storage, the long-term stability of the functionalized particles, as well as the durability of their antimicrobial properties and capacity to limit histamine formation throughout the shelf life of the product, should be specifically assessed in future storage-stability studies.

## Figures and Tables

**Table 1:** MIC and MBC values of THY and EUG for all studied strains (expressed as mg/mL).

	THY		EUG	
Strains	MIC	MBC	MIC	MBC
*R. planticola*	0.3	0.4	1	1.1
*L. reuteri*	1	1.1	3.5	3.5
*P. parvulus*	1.5	4	2	4
*L. hilgadii*	1	2	2	2.5

**Table 2:** Zeta potential, degree of functionalization (α), and APTES and EOC content and surface coverage for bare and functionalized particles (thymol and eugenol). Values are expressed as mean ± standard deviation (n = 3). Different letters within the same column indicate significant differences among samples (*p* < 0.05).

	Zeta Potencial (mV)	α (mg OM/g SiO_2_)	mg APTES/g SiO_2_	mg EOC/g SiO_2_	APTES-Relative Coverage (%)	EOC Covering (%)
SiO_2_	−27.7 ± 2.2	-	-	-	-	-
SiO_2_-THY	+0.1 ± 1.0 ^a^	136.9 ± 2.4 ^a^	110.5 ± 1.8 ^a^	26.4 ± 1.3 ^a^	37.5 ± 1.2 ^a^	4.5 ± 0.4 ^a^
SiO_2_-EUG	+3.8 ± 1.2 ^b^	139.1 ± 1.1 ^a^	111.0 ± 1.6 ^a^	28.2 ± 1.8 ^a^	39.2 ± 1.0 ^a^	4.5 ± 0.6 ^a^

**Table 3:** Reduction in histamine formation after applying treatment with immobilized compounds.

		Histamine Relative Reduction (%)	
		*R. planticola*	*L. reuteri*
SiO_2_-THY	MIC×2	100.0 ± 0.2 ^Aa^	99.5 ± 0.2 ^Aa^
	MIC	98.1 ± 0.1 ^Aa^	91.2 ± 0.1 ^Bb^
	MIC×0.5	94.8 ± 0.1 ^Aa^	61.1 ± 0.4 ^Bb^
	MIC×0.25	69.5 ± 0.2 ^Aa^	59.8 ± 0.4 ^Aa^
SiO_2_-EUG	MIC×2	98.7 ± 1.0 ^Aa^	100.0 ± 0.0 ^Aa^
	MIC	90.5 ± 0.1 ^Bb^	100.0 ± 0.0 ^Aa^
	MIC×0.5	87.5 ± 0.1 ^Bb^	99.4 ± 0.1 ^Aa^
	MIC×0.25	82.2 ± 0.1 ^Aa^	92.3 ± 0.2 ^Aa^

Values are expressed as mean ± standard deviation (n = 3). Different uppercase letters indicate significant differences between compounds at the same concentration and bacterial strain, while different lowercase letters indicate significant differences between bacterial strains within the same treatment (*p* < 0.05).

## Data Availability

The original contributions presented in this study are included in the article/Supplementary Materials. Further inquiries can be directed to the corresponding author.

## Supplementary Materials

The following supporting information can be downloaded at: https://www.mdpi.com/article/10.3390/foods15122067/s1, Table S1: Validation parameters of the chromatographic analysis for the histamine determination; Figure S1: HPLC chromatograms used for histamine analysis; Figure S2: Representative images of microbial colony counts.
