# Supplementary material for: Silica Particles Functionalization with Thymol and Eugenol as a Novel Strategy to Control Histamine-Producing Bacteria
Source: Foods. 2026 Jun 8;15(12):2067. doi: 10.3390/foods15122067 (PMC13297833; doi:10.3390/foods15122067)
Supplement: Supplementary file 1 [file foods-15-02067-s001.zip › foods-4350115-supplementary.pdf]

# Silica Particles Functionalized with Thymol and Eugenol as a Novel Strategy to Control Histamine-Producing Bacteria

**Oumaima Moumane[1], Alejandro Rivas Soler [1], Ana Fuentes López[1], José Manuel Barat Baviera[1], Édgar Pérez Esteve[1]*.**

**Table S1.** Validation parameters of the chromatographic analysis for the histamine determination.

| Equation | Linearity ($R^2$) | Range (mg/L) | LOD (mg/L) | LOQ (mg/L) | Precision (%RSD) | | Accuracy (%) | RECOVERY (%) |
|---|---|---|---|---|---|---|---|---|
| | | | | | Intra-day | Inter-day | | |
| **Y = 290753X + 29217** | 0.9999 | 0.1 – 50 | 0.37 | 1.14 | 7.93 | 9.30 | 100.75 | 107.02 |

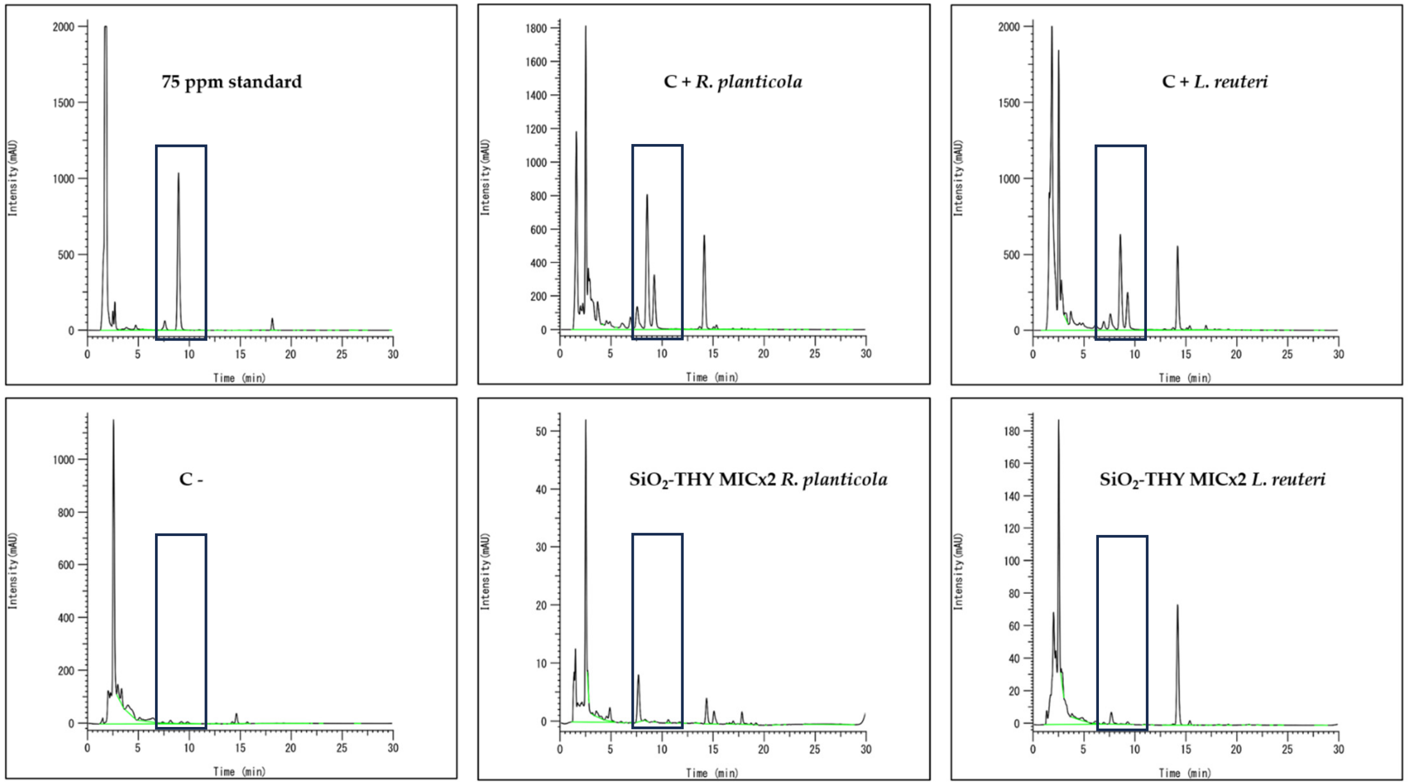


Figure S1. HPLC chromatograms used for histamine analysis

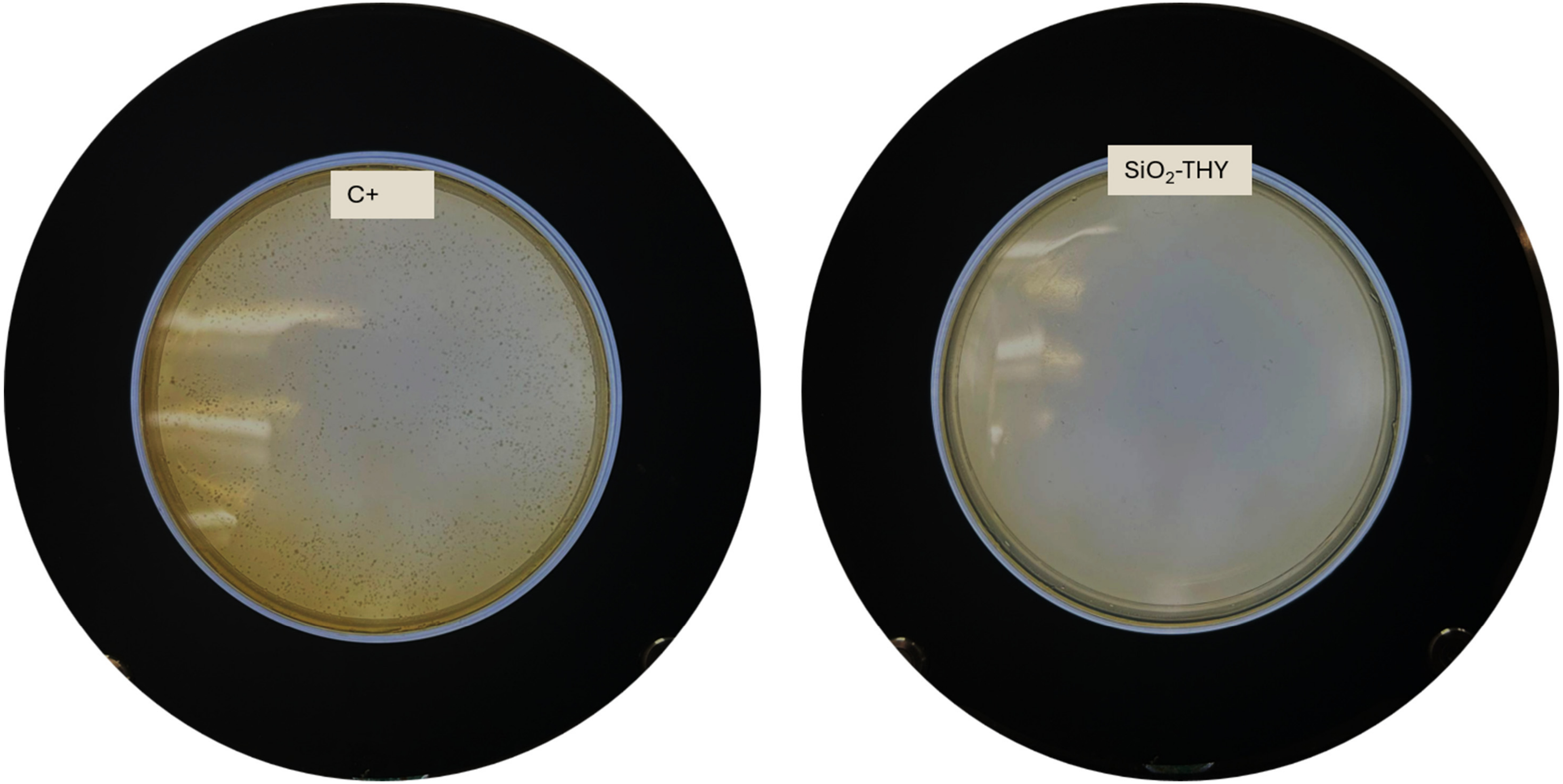


Figure S2. Representative images of microbial colony counts. C+: Growth of *L. Reutery*. $SiO_2$-THY: Growth of *L. Reuteri* in the presence of $SiO_2$-THY.
